# Laparoscopic Exploration Converted to Laparotomy in a Case of Rectal Perforation and Peritonitis After Administration of Enema

**DOI:** 10.7759/cureus.21211

**Published:** 2022-01-13

**Authors:** Hany A Zaki, Adel Zahran, Ahmed E Shaban, Haris Iftikhar, Eman E Shaban

**Affiliations:** 1 Emergency Medicine, Hamad Medical Corporation, Doha, QAT; 2 Internal Medicine, Mansoura General Hospital, Mansoura, EGY; 3 Internal Medicine, Faculty of Medicine, Mansoura University, Mansoura, EGY; 4 Cardiology, Al Jufairi Diagnostics and Treatment, Doha, QAT

**Keywords:** rectal perforation, phosphate enema, laparotomy, laparoscopic exploration, peritonitis, laparoscopy

## Abstract

Laparoscopy is the preferred surgical approach to several ailments because it permits accurate and efficient diagnosis and treatment. In abdominal conditions, the clinician may accomplish both treatment components - exploration for identification of the causative pathology and the conduction of a proper operation - through laparoscopy. There is an ongoing debate of peritonitis as a contraindication to this approach. Laparoscopy has been widely accepted in many subspecialties over the last decade. Peritonitis is usually treated by a conventional open surgery which has a significantly high morbidity and mortality rate. In the present case, a 24-year-old male presented to the emergency unit of our facility with constipation for the past week, along with the inability to urinate with suprapubic pain and tenderness. The patient developed severe abdominal pain within an hour of receiving an enema injection. The patient was started on broad-spectrum antibiotics due to suspected peritonitis and later sent for imaging studies with urgent surgical consultation. The patient remained in the hospital for a few days and was then discharged after a week of hospital administration. It is worth mentioning that therapeutic laparoscopy may be considered in select cases of abdominal trauma. It offers more advantages over laparotomy, including reduced complication rate, length of stay, and mortality.

## Introduction

Peritonitis is a significant contributor to surgical emergency and intervention. The wide acceptance of laparoscopy due to specific advantages such as short hospital stays, less pain, and decreased morbidity [1-4] is a primary reason why surgeons apply it in cases where it was previously contraindicated.

Laparoscopy has been employed in the management of gastrointestinal conditions such as colonic perforations and peptic ulcers way back in the 1990s [2, 3]. Apart from its recognized role in elective surgery of the upper gastrointestinal tract, attempts are being made to employ it in cases of generalized peritonitis associated with severe physiological disturbances [5, 6]. Several reports suggest that laparoscopic peritoneal lavage can be performed effectively and that perforations can be closed without complications [7-10]. And so, with the encouraging results that we found in these studies, we present this case report in order to verify the benefit of minimally invasive surgery in gastrointestinal conditions with generalized or localized peritonitis.

## Case presentation

A 24-year-old male patient presented to the emergency unit of our facility with constipation that had affected him over the past week. The patient passed hard stool with difficulty and also reported an inability to urinate with suprapubic pain. The patient also experienced tenderness for the last nine hours prior to arrival at the emergency department. He, however, denied nausea or vomiting. There were also no reports of respiratory disturbances or fever, no shortness of breath, chest pain, or dizziness. The patient denied any recent hospital admission or previous surgical intervention. The patient had a social history of smoking and occasional use of illicit drugs (used opioids in his country of origin). There was no family history of chronic illness.

A clinical decision was taken to catheterize the patient and give him glycerin suppositories and phosphate enema, as well as abdominal X-ray, pain management, urine and blood investigations, and then for reassessment.

An hour after receiving the phosphate enema injection, the patient developed severe abdominal pain with diffuse guarding and rigidity. The pain was referred to the right anterior shoulder highlighting the possibility of peritonitis (the patient described the character of the pain as blasting in his abdomen). All vital signs were normal as follows: temperature (oral) at 37.5 °C; peripheral heart rate at 143 beat/minute; respiratory rate, 24 breath/minute; SBP - 148 mmHg; DBP - 97 mmHg; and SpO2 - 100%.

**Figure 1:**
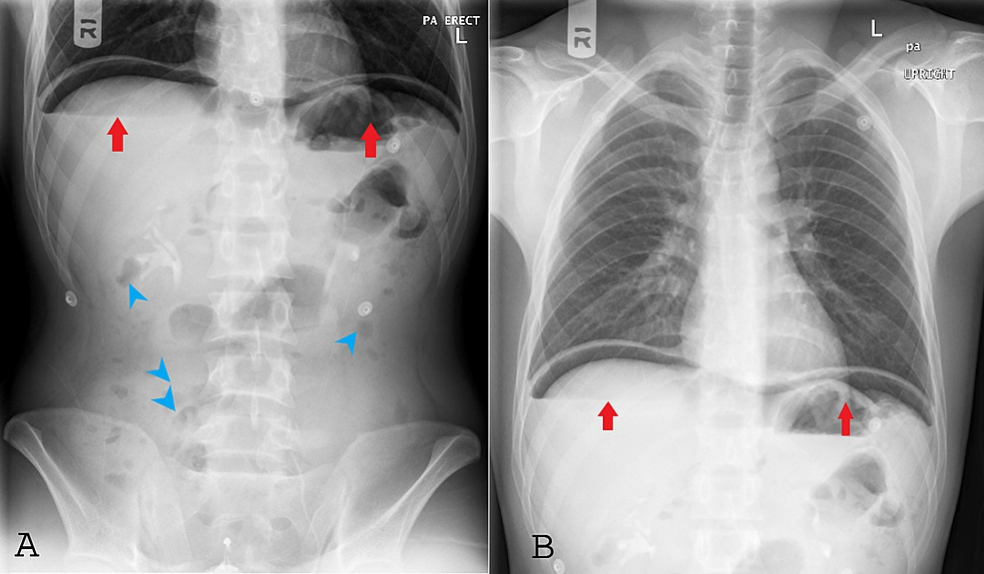
Abdominal X-ray (left side) and chest X-ray (right side) (A) Abdominal X-ray (left side) showed bowel gas pattern is within normal limits, with no dilated bowel loops / significant air-fluid levels. Fecal residue along the course of the colon (blue arrowheads) with significant air under the diaphragm on both sides (red arrows). Psoas outlines appear normal. (B) Chest X-ray (right side) showed clear costophrenic angles and lung fields. Mediastinum and hila appear normal. The cardiac size and silhouette appear to be within normal limits. The pneumoperitoneum is indicated with red arrows.

**Figure 2:**
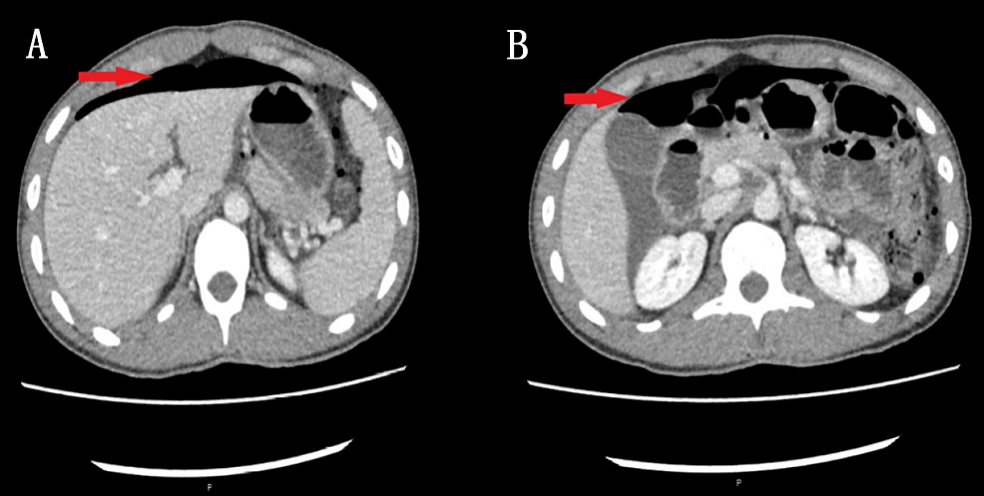
Computed tomography abdomen (A) and pelvis (B) with contrast axial sections Imaging showed extensive pneumoperitoneum with generalized ascites (red arrows).

**Figure 3:**
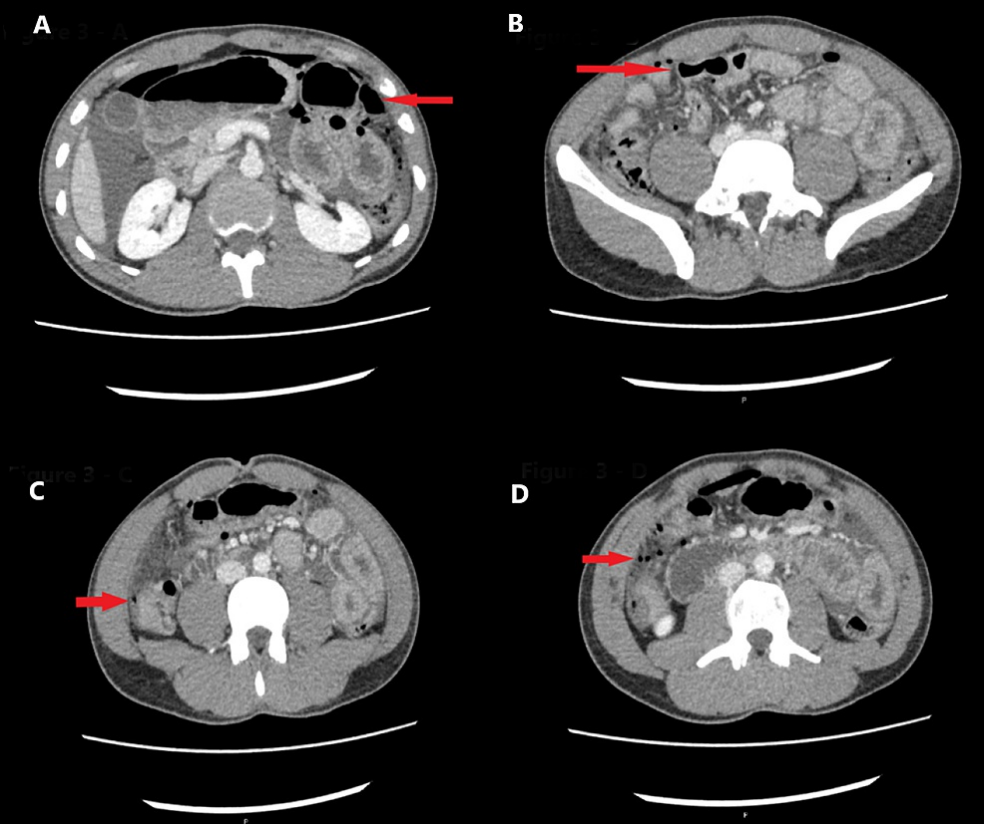
(A) showed air in the mesenteric fold of splenic flexure, (B) showed extensive pneumoperitoneum (red arrow), (C) showed omental thickening in the right lumbar region, and (D) showed specks of air in mesenteric folds of the right iliac region and adjoining hepatic and splenic flexures

Treatment planned to shift the patient to the resuscitation room due to his sickly appearance with a high suspicion of acute abdomen with suspected perforation. The patient was then connected to the cardiac monitor and started on intravenous morphia titrating to the pain score. We started broad-spectrum antibiotics intravenous (tazocin) due to suspected peritonitis and later sent for imaging for both chest X-ray, and abdominal X-ray (Figure 1). After reviewing the chest and abdomen X-ray, we found evidence of air under the diaphragm on both sides, then ordered an abdominal CT with contrast (Figures 2-3); after this, immediate urgent surgical consultation was done.

Then the surgeon on call decided to send the patient to the theatre for diagnostic laparoscopy and proceed with possible conversion laparotomy under general anesthesia. The plan of treatment and possible complications were discussed with the patient who willingly gave his consent.

Looking at the findings, there was fecal soiling of the abdominal cavity. Running along the bowel was a 2 cm perforation of the anterior wall of the upper rectum with ecchymotic edges of the perforation site. There was no palpable mass, and the large bowel was not loaded with stool. We perforated the edges of the rectal side for histopathology. Abdominal fluid was aspirated for culture.

Technique

The patient was placed in a supine position under the aseptic technique under general anesthesia. We did a 10mm supraumbilical incision using blade 11 by open technique. CO2 was inflated into the pneumoperitoneum till 15 mmHg. A 10 mm trocar and camera were inserted. Two 5 mm trocars were inserted into the left and right umbilicus. After consulting with the colorectal surgeon, they attended the theatre and advised for conversion to open and to go with primary repair and loop sigmoidal colostomy. After refreshing the edges, the primary closure was done with Vicryl 2/0 and the second layer of 2/0 Vicryl. After irrigation with 6 liters of normal saline, lateral dissection of the sigmoid colon and descending colon and loop was able to reach freely to the abdominal wall.

The stoma opening was created at semilunaris to the left side. Linea alba closed with polydioxanone suture (PDS) loop, and the skin closed with skin clips. The loop was fixed to the sheath, with Vicryl 3/0, and to the skin 3/0 Vicryl 3/0. Digital rectal examination showed no palpable mass.

Post-operative steps

The patient was intubated with a nasogastric tube and Foley's urinary catheter, painkillers, judicious hydration, IV antibiotic, and antiemetics. The patient was admitted to the ordinary ward after clearance and assessment were done from the ICU.

A specimen from the perforated rectum wall was sent to the pathology and it consisted of two mucosal fragments, measuring 0.8 - 0.9 cm, with the results showing transmural necrosis and acute inflammation, consistent with perforation.

**Figure 4:**
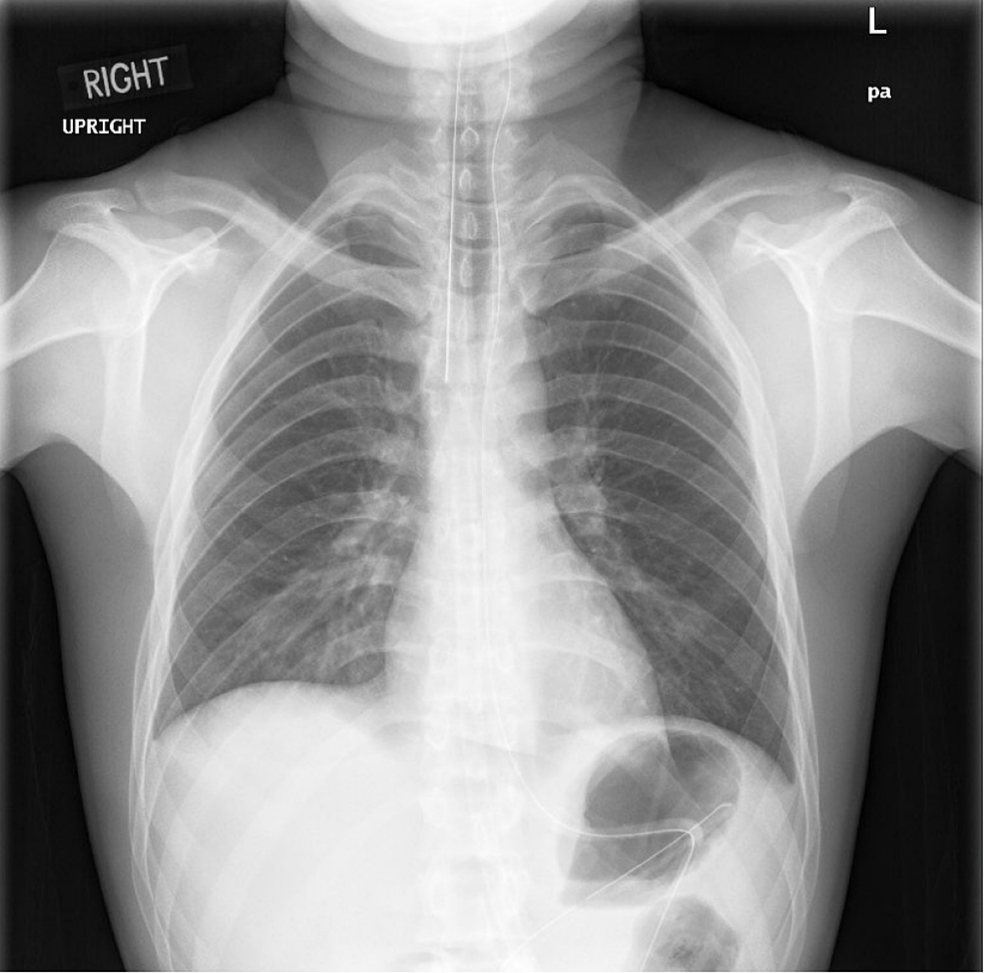
Postoperative chest X-ray Chest X-ray a few days post-operative after admission showed no abnormalities with significant complete resolution of the pneumoperitoneum bilaterally.

The patient stayed for a few days in the hospital and a chest X-ray was done again for follow-up which showed no abnormalities (Figure 4), and then was discharged within a week of hospital admission.

## Discussion

Exploratory laparotomy has been the conventional treatment for penetrating abdominal trauma. Despite the high accuracy and versatility for diagnosing and treating such injuries, some patients have no underlying abdominal injuries resulting in a nontherapeutic laparotomy (NL). These are linked with unnecessary complications in at least 41% of patients [11, 12]. In a more recent study, Zaki et al. stated that inguinal hernia is the commonest complication that affects the anterior abdominal wall, further explaining that increased intra-abdominal pressure is a primary risk factor of inguinal hernia formation [13]. In another large data study, the researchers found that nontherapeutic laparotomy was associated with higher mortality when compared to diagnostic laparoscopy [14] (OR 4.5), a high rate of complications (OR 2.2), and a prolonged hospital stay (OR 2.7). Nontherapeutic laparotomy was also associated with high rates of pneumonia, acute respiratory distress syndrome, myocardial infarction, and venous thromboembolism [14]. The contrast appears similar when comparing therapeutic laparotomy to therapeutic laparoscopy in patients positive for abdominal injury. In a study involving 518 patients, Chestovich and colleagues found that length of stay was shorter in the therapeutic laparoscopy group compared to that in the therapeutic laparotomy group (four days and two days, respectively). There were more cases of wound infections with open exploration (10.4% vs. 0%), as was the development of small bowel obstruction or ileus (1.1% vs. 9.4%) [15].

For laparoscopy to be used in traumatic injury treatment, it must be efficient, safe, and reliable for diagnostic purposes and also provide therapeutic value in selected patients. While some studies have reported laparoscopic exploration and treatment for traumatic injuries, there is some level of hesitancy among the trauma community to accept it. This stems from a couple of factors, such as early reports of missed injuries, inability to visualize various regions of the abdomen, increased operative time, which are of immense importance during periods of high trauma volume [16].

However, there is an increase in advanced training in minimally invasive surgery, and this is believed to help mitigate the burden that may plague trauma specialists.

## Conclusions

Based on our experience, laparoscopy in the therapeutic management of abdominal emergencies due to peritonitis is undoubtedly a possibility as it is simple, reproducible, and also effective without complications in experienced hands; it also has higher accuracy and a wide therapeutic potential. Not subjecting patients to unnecessary laparotomies minimizes postoperative pain, increases recovery of gastrointestinal functions, reduces the rate of hospitalization, increases cosmesis, and contains healthcare costs.
